# Interventions to improve birth outcomes of pregnant women living in low- and middle-income countries: a systematic review and network meta-analysis

**DOI:** 10.12688/gatesopenres.13081.2

**Published:** 2020-09-24

**Authors:** Jay J. H. Park, Ofir Harari, Ellie Siden, Michael Zoratti, Louis Dron, Noor-E Zannat, Richard T. Lester, Kristian Thorlund, Edward J. Mills

**Affiliations:** 1 MTEK Sciences, Vancouver, BC, V5Z 1J5, Canada; 2 Experimental Medicine, Department of Medicine, University of British Columbia, Vancouver, BC, V5Z 1M9, Canada; 3 Department of Health Research Methodology, Evidence, and Impact, McMaster University, Hamilton, Ontario, L8S 4L8, Canada

**Keywords:** Pregnancy, low- and middle-income countries, network meta-analysis, evidence synthesis, preterm, birthweight, birth outcomes

## Abstract

**Background: **Improving the health of pregnant women is important to prevent adverse birth outcomes, such as preterm birth and low birthweight. We evaluated the comparative effectiveness of interventions under the domains of micronutrient, balanced energy protein, deworming, maternal education, and water sanitation and hygiene (WASH) for their effects on these adverse birth outcomes.

**Methods: **For this network meta-analysis, we searched for randomized clinical trials (RCTs) of interventions provided to pregnant women in low- and middle-income countries (LMICs). We searched for reports published until September 17, 2019 and hand-searched bibliographies of existing reviews. We extracted data from eligible studies for study characteristics, interventions, participants’ characteristics at baseline, and birth outcomes. We compared effects on preterm birth (<37 gestational week), low birthweight (LBW; <2500 g), and birthweight (continuous) using studies conducted in LMICs.

**Results: **Our network meta-analyses were based on 101 RCTs (132 papers) pertaining to 206,531 participants. Several micronutrients and balanced energy food supplement interventions demonstrated effectiveness over standard-of-care. For instance, versus standard-of-care, micronutrient supplements for pregnant women, such as iron and calcium, decreased risks of preterm birth (iron: RR=0.70, 95% credible interval [Crl] 0.47, 1.01; calcium: RR=0.76, 95%Crl 0.56, 0.99). Daily intake of 1500kcal of local food decreased the risks of preterm birth (RR=0.36, 95%Crl 0.16, 0.77) and LBW (RR=0.17, 95%Crl 0.09, 0.29), respectively when compared to standard-of-care. Educational and deworming interventions did not show improvements in birth outcomes, and no WASH intervention trials reported on these adverse birth outcomes.

**Conclusion: **We found several pregnancy interventions that improve birth outcomes. However, most clinical trials have only evaluated interventions under a single domain (e.g. micronutrients) even though the causes of adverse birth outcomes are multi-faceted. There is a need to combine interventions that of different domains as packages and test for their effectiveness.

**Registration: **PROSPERO CRD42018110446; registered on 17 October 2018.

## Introduction

Despite global substantial progresses that have been made towards improving maternal, newborn, and child health (MNCH) in the last two decades, adverse birth outcomes such as preterm birth and low birthweight still remain as an important global health challenge, particularly in low- and middle-income countries (LMIC) ^1– 3^. Determinants of these challenges are multifaceted ^4– 7^. Pregnant women in LMICs have a higher risk of nutritional deficiencies, stemming from physiological changes that involve fetal development and growth resulting in an increased demand for nutrients ^4, 5^. Poor water, sanitation, and hygiene (WASH) control can also increase likelihood for infectious diseases, including intestinal worm infections that may contribute to conditions, such as anemia, which negatively affects fetal survival and growth ^6, 8, 9^. Poor maternal health during pregnancy is associated with preterm birth (<37 gestation weeks) and low birthweight (<2500 g), and these adverse birth outcomes are associated with adverse neonatal events, such as respiratory distress syndrome, neurocognitive impairment, poor linear growth (stunting), and overall mortality ^1, 2, 10, 11^.

Several reviews have aimed to assess the effectiveness of various promising interventions for pregnant women ( Table 1). Despite the extensive research conducted to date, the comparative effectiveness of interventions remains unclear across different domains, such as micronutrients, balanced energy protein supplements, maternal education, deworming, and WASH. Few clinical trials have directly compared interventions across domains. Rather, the majority of clinical trials has only compared interventions within a domain. Similarly, most summaries of the evidence for pregnancy interventions have used traditional pairwise meta-analysis, allowing only for the quantitative assessment of a single intervention versus a comparator. Thus far, no attempts have been made to synthesize the evidence indirectly in order to make quantitative comparison of interventions that have not been directly compared in studies.

**Table 1.** Existing reviews on interventions for pregnant women.

Review ID	Title	Interventions	No of studies	Types of studies included
Imdad 2011 ^20^	Effect of balanced protein energy supplementation during pregnancy on birth outcomes	Balanced protein energy supplements	11	RCTs and quasi-RCTs
Imdad 2012 ^21^	Maternal Nutrition and Birth Outcomes: Effect of Balanced Protein-Energy Supplementation	Balanced protein energy supplements	16	RCTs and quasi-RCTs
Liberato 2013 ^22^	Effects of protein energy supplementation during pregnancy on fetal growth: a review of the literature focusing on contextual factors	Balanced protein energy supplements	20	RCTs, quasi-RCTs, and observational study
Stevens 2015 ^23^	The effect of balanced protein energy supplementation in undernourished pregnant women and child physical growth in low- and middle-income countries: a systematic review and meta-analysis	Balanced protein energy supplements	7	RCTs, quasi-RCTs, and observational study
Buppasiri 2015 ^24^	Calcium supplementation (other than for preventing or treating hypertension) for improving pregnancy and infant outcomes	Calcium	25	RCTs and cluster-RCTs
Hofmeyr 2014 ^25^	Calcium supplementation during pregnancy for preventing hypertensive disorders and related problems	Calcium	13	RCTs
Salam 2015 ^26^	Effect of administration of antihelminthics for soil-transmitted helminths during pregnancy	Deworming	4	RCTs
Lassi 2013 ^27^	Folic acid supplementation during pregnancy for maternal health and pregnancy outcomes	Folic acid	31	RCTs and cluster-RCTs
Yang 2011 ^28^ *	Review of fortified food and beverage products for pregnant and lactating women and their impact on nutritional status	Fortified products	14	RCT, quasi-RCT
Pena-Rosas 2009 ^29^	Effects and safety of preventive oral iron or iron+folic acid supplementation for women during pregnancy	Iron; Iron + folic acid	49	RCTs and quasi-RCTs
Suchdev 2015 ^30^	Multiple micronutrient powders for home (point-of-use) fortification of foods in pregnant women (Review)	Multiple micronutrient powders	2	RCTs
Haider 2017 ^31^	Multiple-micronutrient supplementation for women during pregnancy	Multiple micronutrient supplements	19	RCTs
Imhoff-Kunsch 2012 ^32^	Effect of n-3 Long-chain Polyunsaturated Fatty Acid Intake during Pregnancy on Maternal, Infant, and Child Health Outcomes: A Systematic Review	N-3 long chain polyunsaturated fatty acid	15	RCT
Thorne-Lyman 2012A ^33^	Vitamin A and carotenoids during pregnancy and maternal, neonatal and infant health outcomes: a systematic review and meta- analysis	Vitamin A	17	RCTs
De-Regil 2016 ^34^	Vitamin D supplementation for women during pregnancy	Vitamin D	15	RCTs and quasi-RCTs
Perez-Lopez 2015 ^35^	Effect of vitamin D supplementation during pregnancy on maternal and neonatal outcomes: a systematic review and meta- analysis of randomized controlled trials	Vitamin D	13	RCTs
Thorne-Lyman 2012B ^36^	Vitamin D during pregnancy and maternal, neonatal and infant health outcomes: a systematic review and meta-analysis	Vitamin D	5	RCTs
Ota 2015 ^37^	Zinc supplementation for improving pregnancy and infant outcome	Zinc	21	RCTs
Goudet 2019 ^38^	Nutritional interventions for preventing stunting in children (birth to 59 months) living in urban slums in low-and middle-income countries (lmic)	Nutrient supplementation and Education	15	RCTs, quasi-RCTs, non- RCTs, controlled before- and-after, and interrupted time series

*All reviews were systematic literature reviews with pairwise meta-analysis, except for Yang 2011.

Recognizing the paucity of direct head-to-head randomized clinical trials (RCTs) between existing interventions, a network meta-analysis can be used to summarize the entirety of evidence for pregnancy interventions. A network of interventions connected via the comparisons that have been made in head-to-head trials can be constructed, and where there is a path from one intervention to another, these interventions can be compared indirectly via some common comparators ^12– 16^. In addition, where both direct and indirect evidence exists, the indirect evidence can be used to strengthen the inferences for the particular comparison. This is particularly important for pregnancy interventions because many head-to-head trials of active interventions have limited sample sizes. Furthermore, network meta-analysis allows us to simultaneously analyze all potential treatment options and make full use of the available evidence within a single analysis.

The purpose of this study was to assess the comparative effectiveness across intervention domains in micronutrient supplements, balanced energy protein supplements, deworming, maternal education, and WASH interventions using network meta-analysis. Effectiveness of interventions are determined by the following outcome indicators: preterm birth, low birthweight, and birthweight for LMIC-based pregnant women.

## Methods

Our analysis and report was designed and reported according to the Preferred Reporting Items for Systematic Reviews and Meta-Analysis (PRISMA) extension to network meta-analysis ^17^. The protocol for this study is registered on PROSPERO ( CRD42018110446).

### Search strategy and selection criteria

The scope of our research study and the corresponding search strategy was developed after first reviewing the papers published in the Lancet’s 2013 Maternal and Child Nutrition series ^1, 18^, including the umbrella review on evidence-based interventions by Bhutta and colleagues ^2^, for an overview of the literature. Specifically, we reviewed the bibliography of Bhutta *et al.* 2013 ^2^ for relevant systematic reviews, global health guidelines, and LMIC-based trials. We also performed hand searches on PubMed and the Cochrane Database of Systematic Reviews for reviews that have been published after 2013. The list of published reviews relevant to this study is provided in Table 1.

For our systematic literature search, the following databases were searched from inception to September 17, 2019: the Cochrane Central Register of Controlled Trials, Embase, and MEDLINE ( *Extended data*, Supplementary Tables 1–3) ^19^. In addition to database searches, we included the relevant trials identified from bibliographies of prior reviews ( Table 1). Table 2 describes the PICOS criteria used to guide the study selection. We included LMIC-based RCTs on interventions related to the domains of micronutrient supplements, balanced energy protein (i.e. food supplementation) supplements, deworming, maternal education, and WASH; the outcomes of interest were preterm birth (<37 weeks of gestational age), low birthweight (<2500 grams), and birthweight (continuous). We excluded non-English language studies.

**Table 2.** Population, interventions, comparator, outcomes, and study design criteria for study inclusion.

Category	Inclusion criteria
Population	Pregnant women living in low- and middle-income countries
Intervention	• Micronutrient and calcium supplementation to mother • Balanced energy protein (i.e. food) supplementation to mother • Deworming • Maternal education • Any water, sanitation and hygiene intervention
Comparators	• Placebo • Standard-of-care (if applicable) • No intervention • Any of the interventions listed above as monotherapy or in combination that can be used for indirect comparison
Outcomes	At least one of the following outcomes: • Preterm birth (<37 weeks of gestational age) • Low birthweight (<2500 g) • Birthweight (continuous)
Study Design	Randomized clinical trials
Other	Published in the English language

A paired group of four reviewers (JJHP, ES, MZ, and LD) independently reviewed all abstracts and proceedings identified in the literature searches. JJHP and ES worked in one pair, while MZ and LD worked in another pair. The same paired team independently reviewed abstracts potentially relevant in full-text. If any discrepancies occurred between the studies selected by the two investigators, a third investigator (KT) provided arbitration.

Using a standardized data sheet, a paired group of four reviewers (JJHP, ES, MZ, and NEZ) independently extracted data for study characteristics, interventions used, patient characteristics at baseline, and outcomes from the final list of selected eligible studies. Any discrepancies observed between the data extracted by the four extractors were resolved by consensus through discussion. Primary outcomes were dichotomous, consisting of preterm birth and low birthweight. Our secondary endpoint was the continuous outcome of birthweight. We preferentially extracted intention-to-treat outcomes.

### Data analysis

We performed our analyses within the Bayesian framework in *R* using the R2WinBUGS v14 ** package ^39, 40^. Bayesian models were performed according to the National Institute for Health and Care Excellence (NICE) in their Technical Support Document 2 (TSD2) ^41^. The network diagrams with respective to the analyzed outcome can be seen in *Extended data*, Supplementary Figures 1–6 ^19^. Estimates of comparative effectiveness are measured using risk ratios (RRs) with associated 95% credible intervals (95% CrI) for preterm birth and low birthweight, and mean differences and the associated 95% CrI for birthweight. We performed random-effects network meta-analysis models using an empirically informative priors for the heterogeneity variance, as suggested by Rhodes *et al.* ^42^ for mean birthweight and Turner *et al.* ^43^ for preterm birth and low birthweight. This was done to stabilize the estimation of heterogeneity in the face of low number of trials per comparison in the network. Our model selection was informed by the deviance information criterion (DIC) and the deviance-leverage plots that could help identify outliers or lack of model fit.

As our primary analysis, we included both cluster and individually randomized (non-cluster) clinical trials. To adjust for clustering effects of the cluster trials, we adjusted the sample sizes and number of cases for preterm birth and low birthweight and inflated variances for mean birthweight to account for clustering effects of the cluster trials, as recommended by Uhlmann *et al.* ^44^, assuming a conservative intra-cluster correlation coefficient (ICC) value of 0.05. For each outcome, we performed sensitivity analyses by excluding cluster randomized clinical trials where the analyses were limited to individually randomized clinical trials only.

### Risk of bias within and across studies

Each full text article was evaluated for reporting quality according to the Cochrane Risk of Bias Tool ^45^. The risk of bias assessment within and across studies are provided in the *Extended data* (Supplementary Table 8) ^19^.

## Results

**Figure 1.**
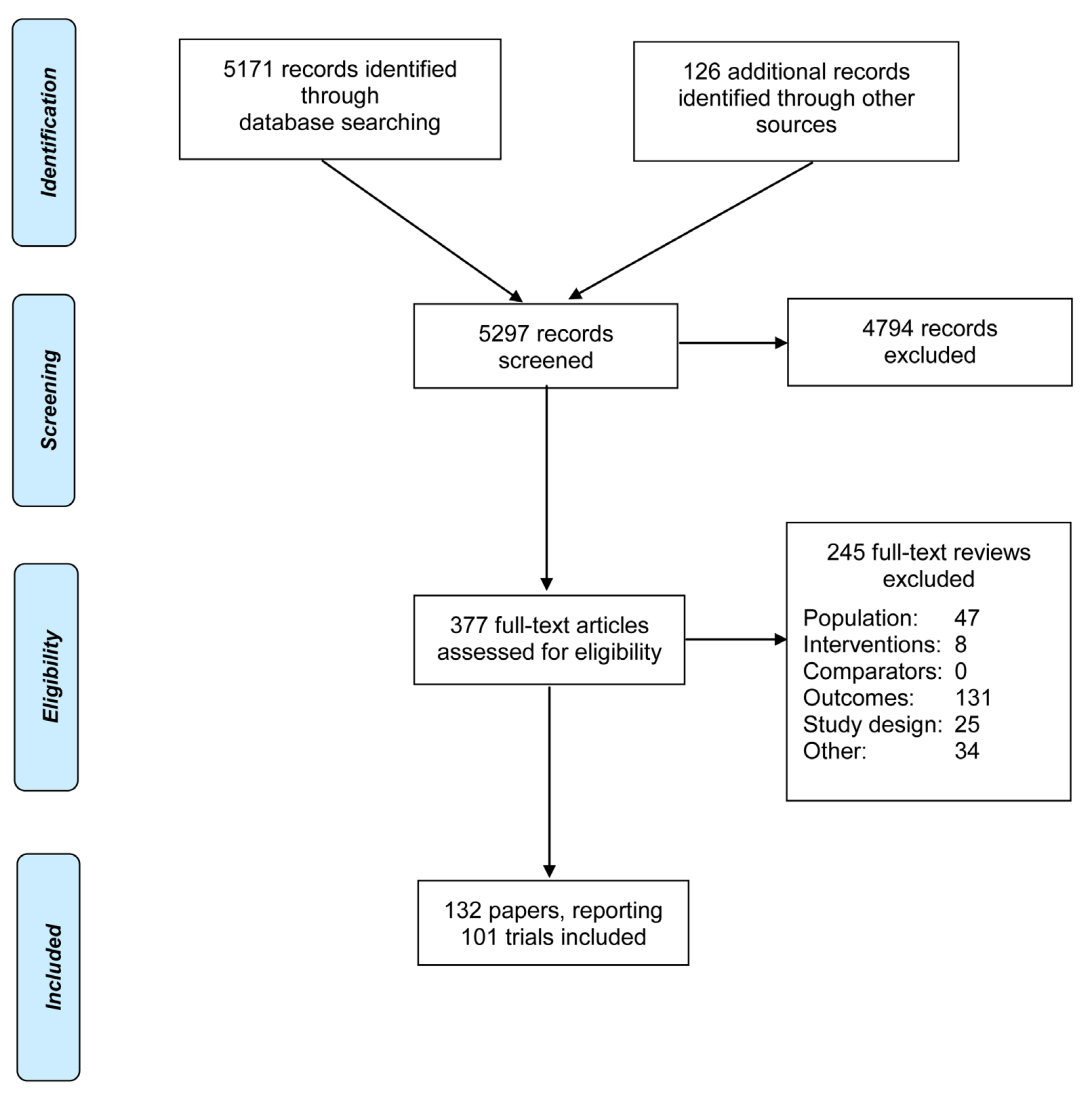
Study selection.

**Figure 2.**
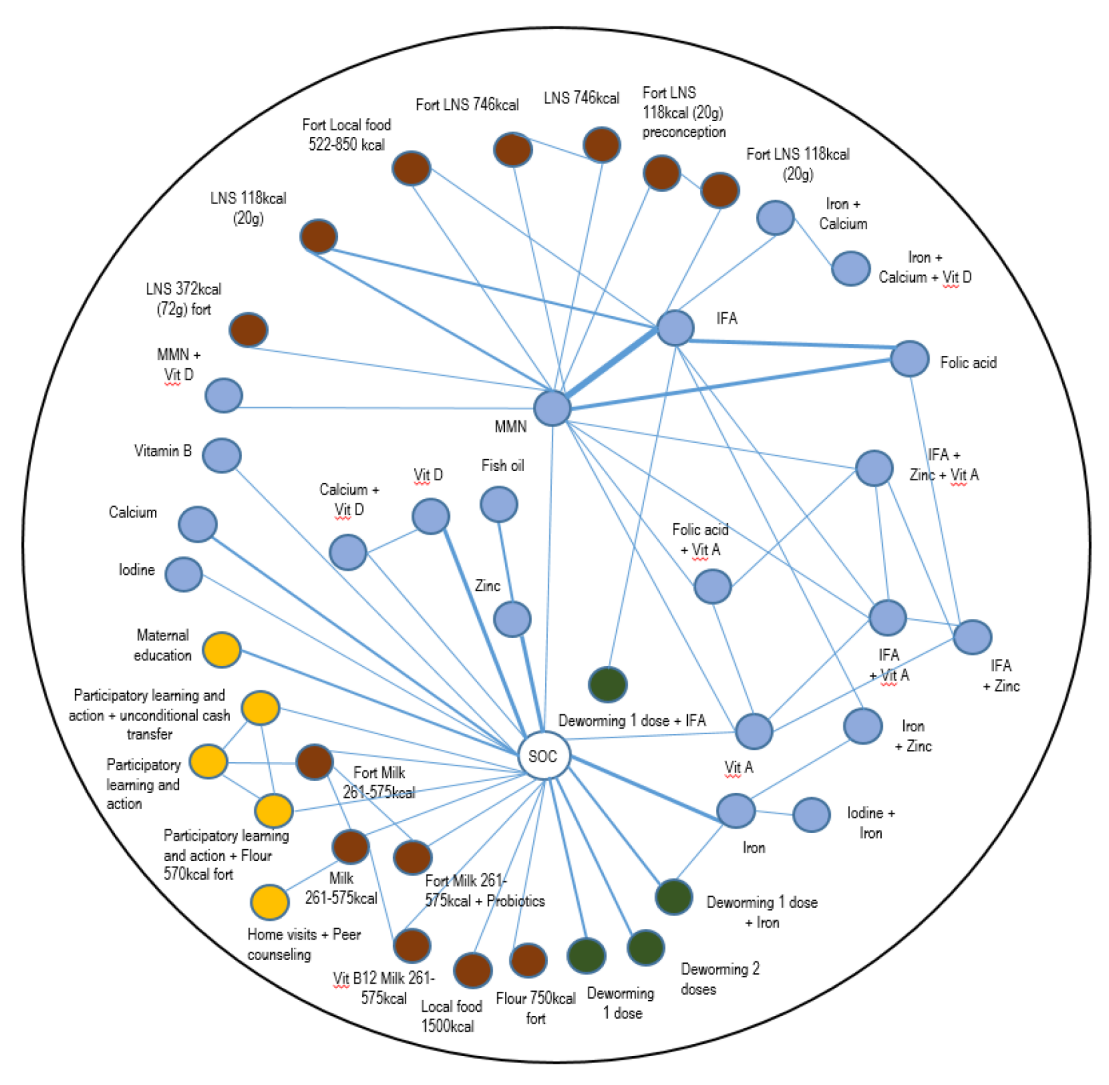
Overall network of the comparisons between interventions for pregnancy. Each node (circle) represents an intervention, each line represents a direct comparison between interventions, with the lines with width representing the number of trials with the direct comparisons in question (i.e. thicker width represents a direct comparison with larger numbers of trials). The different intervention domains are indicated with the following colors: blue for micronutrient supplements; brown for balanced energy protein supplements; yellow for education and counseling interventions; and green for deworming interventions. Vit, Vitamin; IFA, iron and folic Acid; LNS, lipid-based nutrient supplements; Fort, fortification; MMN, multiple micronutrients.

We identified 5,297 abstracts from our database searches and hand searches of the bibliography of the published reviews ( Figure 1). Of these, 377 studies underwent a full-text review, and 132 papers reporting on 101 trials met our inclusion criteria. In total, these trials included 206,531 pregnant women that were randomized to 245 unique interventions ( Figure 2). The list of included and excluded studies ( *Extended data*, Supplementary Tables 4 and 5) ^19^, as well as the trial and patient characteristics of the included studies ( *Extended data*, Supplementary Tables 6 and 7) ^19^ are provided in the *Extended data*. Geographically, most trials were conducted in South Eastern Asian (n = 38 trials) and African (n = 26 trials) countries, with individual randomization (i.e. non-cluster trials, n = 85 trials) and double blinding (n = 52 trials) being the most common methodological features. Micronutrient supplements was the most common intervention domain that was investigated (n = 79 trials); only a few of these micronutrient trials compared interventions from other domains, such as balanced energy protein supplements (n = 15 trials) and deworming (n = 6 trials). Maternal education was captured in a few trials (n=3) where the education component was in the form of counseling, lifestyle education, and participatory learning action (PLA) with government-mandated women’s groups. PLA involved awareness of the problem of LBW and malnutrition, and strategies to overcome barriers to improved health and nutrition. There were no WASH trials reporting on the analyzed birth outcomes.

In most trials, interventions were provided to pregnant women from enrollment until delivery (n = 87 trials). These trials generally involved women who were in the later part of their gestational age. For instance, only 5 trials enrolled women from or before conception (Owens ^46^, The women First Trial ^47^, CAP Trial ^48^, PRECONCEPT ^49^, and Brabin ^50^), while the majority of trials recruited women who were in the later trimesters, such as the 2 ^nd^ and 3 ^rd^ trimesters (n = 69 trials).

### Preterm birth (<37 weeks of gestational age)

**Figure 3.**
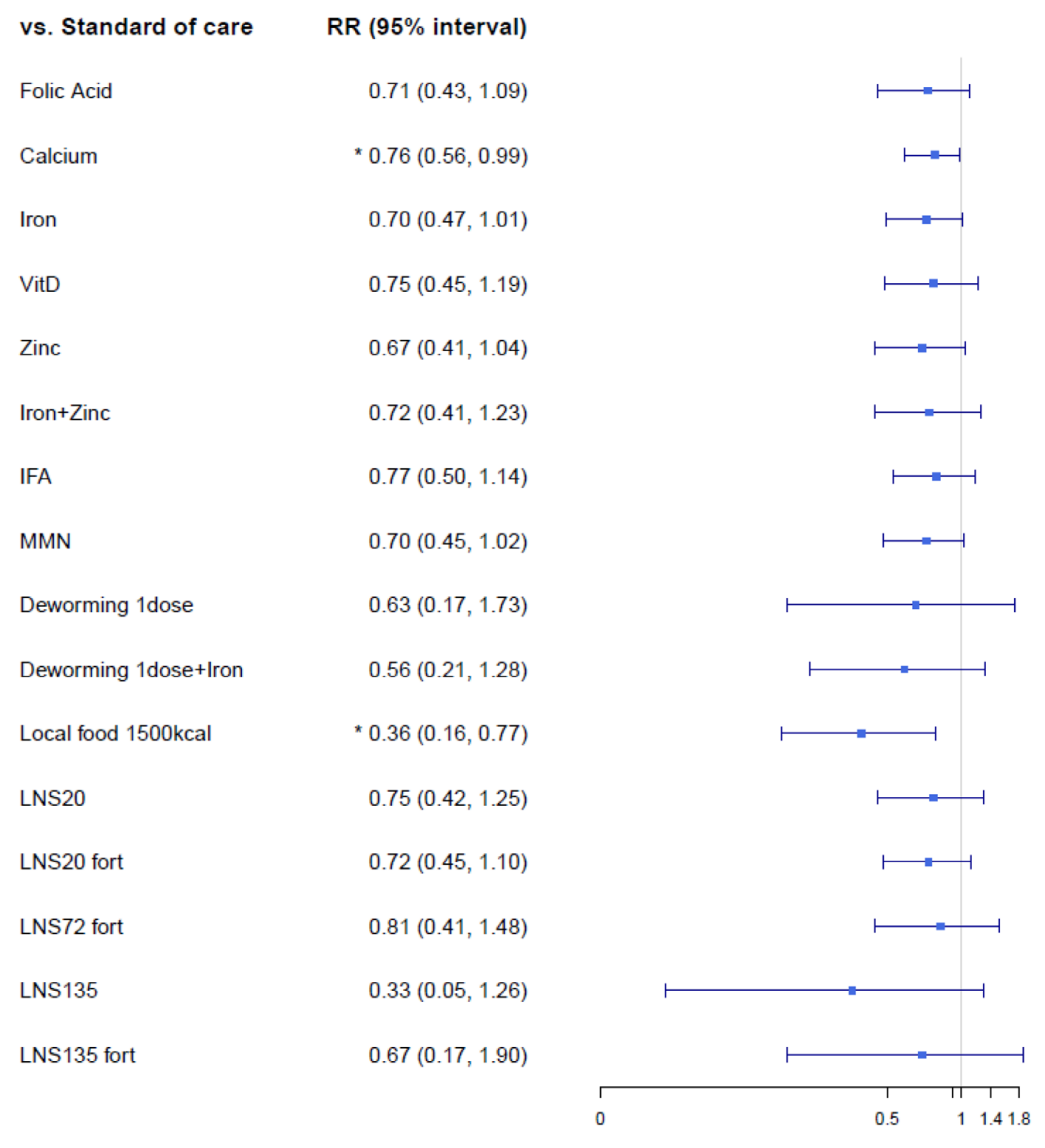
Forest plot for the effects of interventions on preterm birth, risk ratio. Vit, Vitamin; IFA, iron and folic Acid; LNS, lipid-based nutrient supplements; Fort, fortification; MMN, multiple micronutrients.

The preterm birth network ( *Extended data*, Supplementary Figure 1) ^19^ included 64 trials consisting of 85,546 pregnant women randomized to 152 intervention arms (ten cluster trials consisting of 1,998 clusters and 20,218 pregnant women). From the primary analysis, that included both cluster and non-cluster randomized clinical trials, only few interventions showed superiority over standard-of-care for preterm birth ( Figure 3). For instance, compared to standard-of-care, intake of 1500 kcal of local food per day showed an RR of 0.36 (95% CrI: 0.16, 0.77) and calcium showed an RR of 0.76 (95% CrI: 0.56, 0.99). Other micronutrient supplements such as folic acid (RR: 0.71, 95% CrI: 0.43, 1.09), iron (RR: 0.70, 95% CrI: 0.47, 1.01), zinc (RR: 0.67, 95% CrI: 0.41, 1.04), and multiple micronutrients (MMN) (RR: 0.70, 95% CrI: 0.45, 1.02) showed a trend towards lower preterm birth risks compared to standard-of-care, but their Crls overlapped the null effect of 1.00. In comparison to standard-of-care, no balanced energy food supplements, other than 1500 kcal of local food showed reduction in preterm birth risks, and neither did maternal education interventions (e.g. participatory learning action ^51^).

### Mean birthweight (kg)

**Figure 4.**
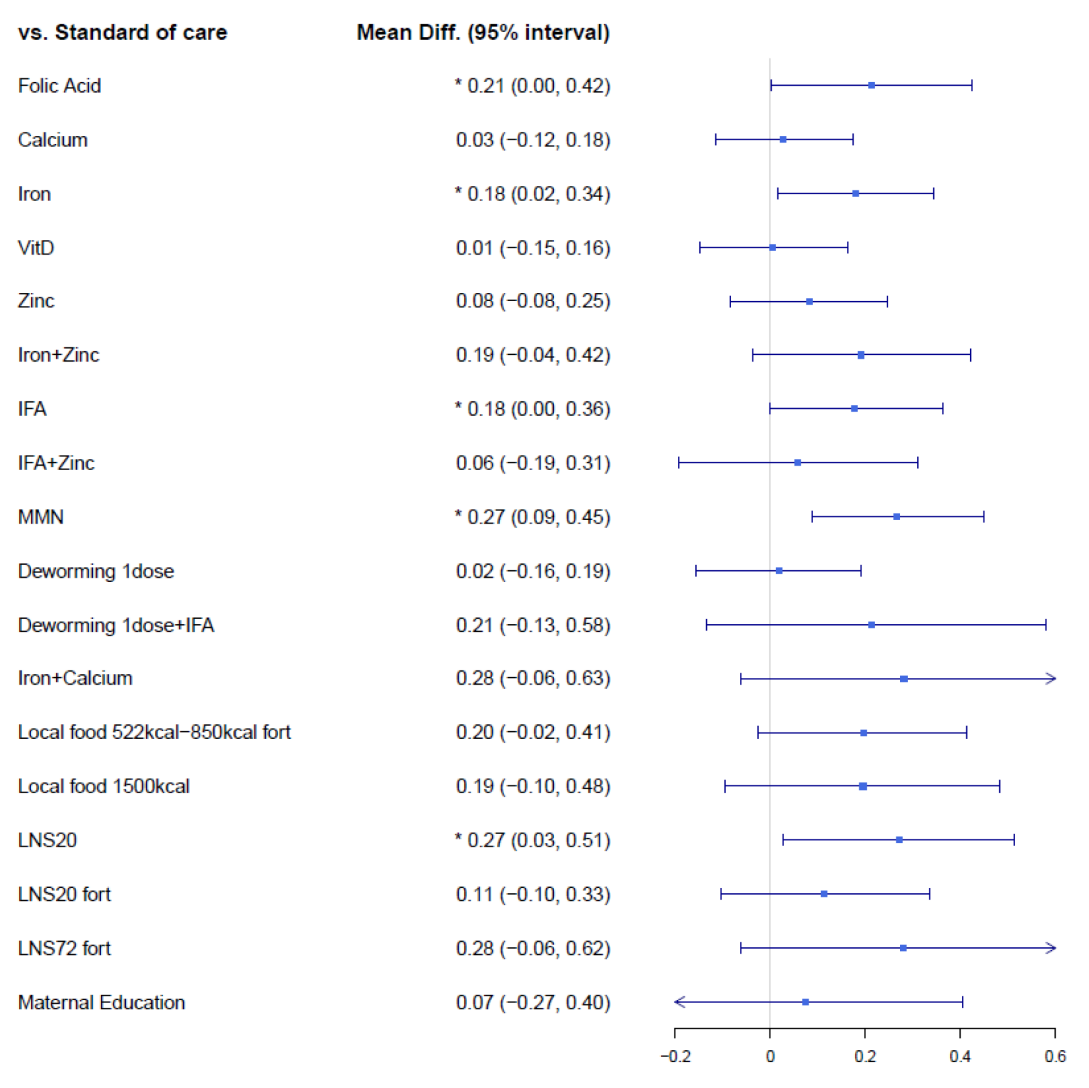
Forest plot for the effects of interventions on birthweight, mean difference in kg. Vit, Vitamin; IFA, iron and folic Acid; LNS, lipid-based nutrient supplements; Fort, fortification; MMN multiple micronutrients.

The mean birthweight network ( *Extended data*, Supplementary Figure 3) ^19^ included of 81 trials that consisted of 130,315 pregnant women randomized to 196 intervention arms. Of these 81 trials, 14 were cluster trials that randomized 1,354 clusters (57,483 pregnant women) to 35 intervention arms. The results of the network meta-analysis on mean birthweight can be found in Figure 4. Among the micronutrient supplementation domain, compared to standard-of-care, MMN (mean difference: 0.27 kg; 95% CrI: 0.09, 0.45 kg), folic acid (mean difference: 0.21 kg; 95% CrI: 0.00, 0.42 kg), iron (mean difference: 0.18 kg; 95% CrI: 0.02, 0.34 kg), and iron + folic acid (IFA) (mean difference: 0.18 kg; 95% CrI: 0.00, 0.36 kg) showed improvements in birthweight. Among the balanced energy food supplements, unfortified lipid-based nutrient supplements of 20 grams (LNS20) showed improvements in birthweight compared to standard-of-care (mean difference: 0.27 kg; 95% CrI: 0.03, 0.51 kg). Deworming and maternal education interventions did not improve mean birth weight; for instance, in comparison to standard-of-care, a single dose of deworming showed a mean difference of 0.02 kg (95% CrI: -0.16, 0.19 kg) and maternal education showed a mean difference of 0.07 kg (95% CrI: -0.27, 0.40 kg).

### Low birthweight (<2.5 kg)

**Figure 5.**
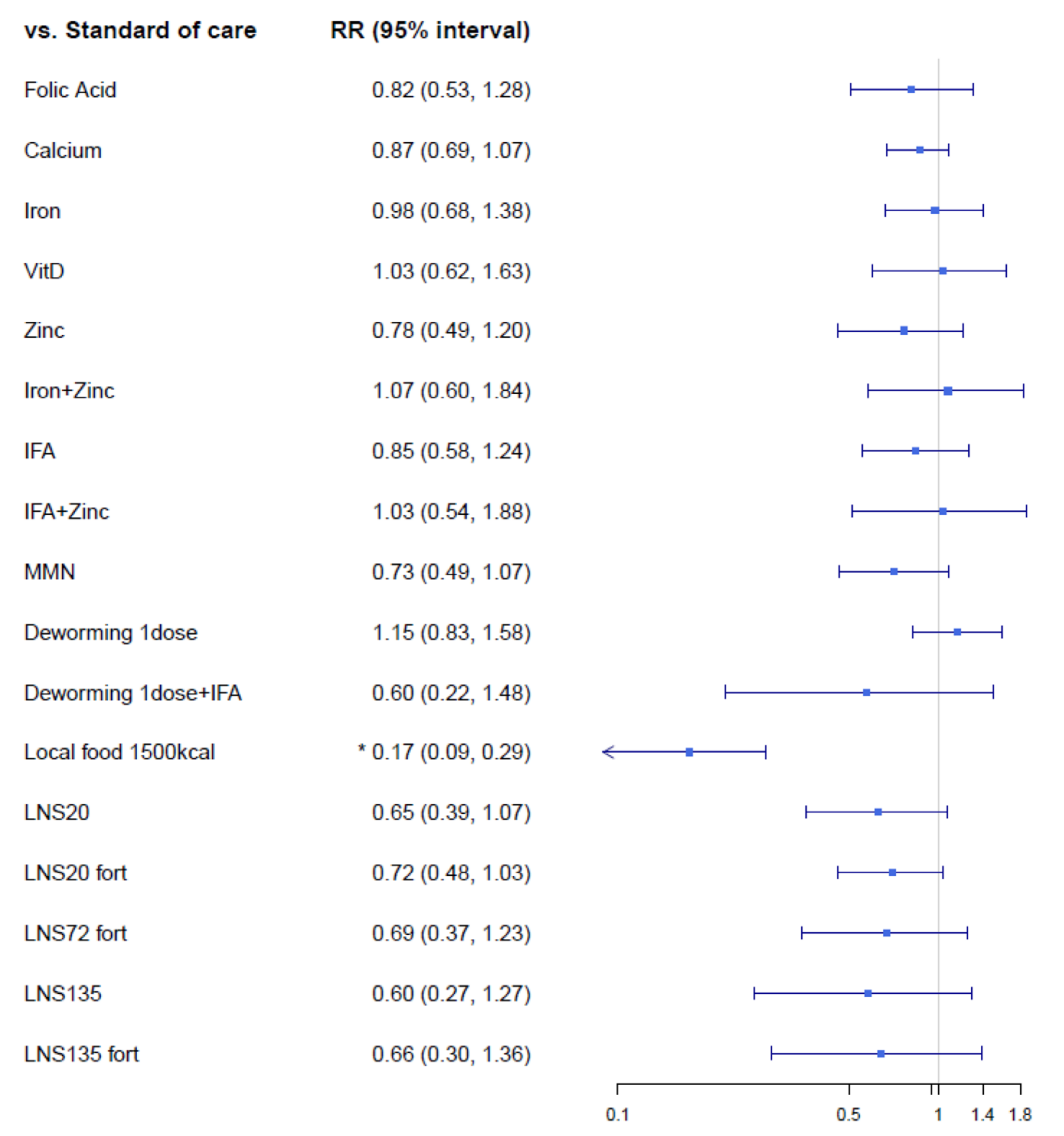
Forest plot for the effects of interventions on low birthweight, risk ratio. Vit, Vitamin; IFA, iron and folic Acid; LNS, lipid-based nutrient supplements; Fort, fortification; MMN multiple micronutrients.

The low birthweight network ( *Extended data*, Supplementary Figure 5) ^19^ consisted of 67 trials, with 84,675 patients randomized to 160 intervention arms (eleven cluster trials consisting of 792 clusters and 9,512 pregnant women). The results on low birthweight (kg) outcome can be found in Figure 5. High caloric local food intervention (1500 kcal per day) reduced the risk of low birthweight (RR: 0.17; 95% CrI: 0.09; 0.29). There was a trend towards reduced risks of low birthweight for other interventions such as calcium (RR: 0.87; 95% CrI: 0.69; 1.07), MMN (RR: 0.73; 95% CrI: 0.49, 1.07), and LNS20 (RR: 0.65; 95% CrI: 0.39, 1.07), fortified LNS20 (RR: 0.72, 95% CrI: 0.48, 1.03), but their 95% CrI contained the null effect of 1.00. A single dose of deworming during pregnancy did not show reduction in low birthweight when compared to standard-of-care (RR: 1.15, 95% CrI: 0.83, 1.58).

### Sensitivity analysis

For all three outcomes, the results from the sensitivity analyses of studies limited to non-cluster randomized clinical trials can be found in the *Extended data* (Supplementary cross table excel file: Sensitivity Preterm, LBW, and Birthweight tabs) ^19^. As fewer studies were available for the sensitivity analysis, the CrIs for many comparisons became wider, but the direction and the magnitude of comparative effects remained relatively stable. For instance, there were no individually randomized trials that evaluated the effectiveness of high caloric (1500 kcal per day) local food. In terms of micronutrient supplementation, MMN (mean difference: 0.10 kg; 95% CrI: 0.00, 0.20 kg) and iron (mean difference: 0.09 kg; 95% CrI: 0.02, 0.16 kg) improved mean birthweight by a small margin compared to standard-of-care. Similarly, unfortified lipid-based nutrient supplements (LNS20) did not improve mean birthweight to a great extent compared to standard-of-care (mean difference: 0.13 kg; 95% CrI: 0.01, 0.24 kg). Furthermore, in comparison to standard-of-care, maternal education (mean difference: 0.04 kg; 95% CrI: -0.13, 0.20 kg) and one dose of deworming (mean difference: 0.01 kg; 95% CrI: -0.04, 0.06 kg) did not have any effect on mean birthweight. As far as preterm birth is concerned, MMN (RR: 0.58; 95% CrI: 0.31, 1.00) showed a trend towards lower preterm birth risks compared to standard-of-care, but their Crls overlapped the null effect of 1.00.

## Discussion

In this study, we used network meta-analysis to compare the effectiveness of interventions across several domains consisting of micronutrient supplements, balanced energy protein supplements, deworming, maternal education, and WASH interventions that can be provided to pregnant women living in LMICs. Several micronutrient supplements demonstrated decreased risks for preterm birth and/or improve mean birthweight, compared with standard-of-care for pregnant women. For example, MMN interventions showed reduction in preterm birth risks and improved mean birthweight. In comparison to standard-of-care, IFA, calcium, iron, and zinc also demonstrated a trend towards decreasing preterm birth risks. However, the evidence for other intervention domains were limited. For instance, among balanced energy protein supplements, only consumption of 1500 kcal of local food supplement lowered the risks of preterm birth and low birthweight; and only unfortified LNS 20 demonstrated improvement in mean birthweight. Nevertheless, these findings pertaining to balanced energy protein supplements corresponded to only three trials in the study ^52– 54^. There was a limited number of trials available for maternal education and deworming intervention; no WASH trials reporting on preterm birth and birthweight outcomes were available.

 The main strength of this study was the use of network meta-analysis to assess the effectiveness of different interventions from a large network of evidence compared to standard-of-care. Unlike previous reviews that have focused on one intervention within a single domain, we used a broad evidence base that included multiple interventions from different domains. As well, appropriate statistical adjustments were made for clustering effects of cluster randomized clinical trials to enable the convergence of cluster and non-cluster trials for our network meta-analysis. Nevertheless, the existing evidence base limited our analyses. Few trials reported low birthweight, and the majority of randomized clinical trial evidence base was confined to a single domain of micronutrient supplementation. Another possible limitation was that there was notable variation in the enrollment of pregnant women in terms of trimesters and gestational age. While we did not find that time of enrollment relative to gestational age was a treatment effect modifier in our analyses, we acknowledge that this variation may have introduced heterogeneity in our meta-analyses. Prior evidence has also demonstrated mixed evidence as to whether the time at which treatment is initiated influences overall treatment efficacy, and this varies by treatment type ^25, 31, 37^. Lastly, our assumption of a conservative ICC (0.05) may also have affected the results. However, this was necessary in order to assess for the entire evidence base of interventions for pregnancy, as most cluster randomized trials did not report ICC for each outcome.

 Despite these limitations, the findings of this study were generally similar to that of other existing reviews. For instance, among the micronutrient supplements, other reviews have shown that iron (RR=0.82, 95%Crl 0.72, 0.94) ^55^ and MMN (RR=0.88, 95%Crl 0.85, 0.90) ^31^ reduced the risks of low birthweight versus standard-of-care. Moreover, calcium (RR=0.76, 95%Crl 0.60, 0.97) ^25^ and zinc (RR=0.86, 95%Crl 0.76, 0.97) ^37^ supplements reduced the risks of preterm birth, and we have found that intake of combined MMN reduced the risks of preterm birth and improved mean birthweight. Similar to this study, Salam ^26^ found no improvements in low birthweight and preterm birth for deworming versus standard-of-care. There were no reviews on WASH available that looked at the role of WASH interventions on birthweight and preterm birth.

Our findings identified several directions for future research. First, there is a need to combine interventions that consist of compelling and evidence-based interventions of different domains as a package, moving away from a reductionist approach that is reflected in the majority of clinical trials conducted so far. Instead of a singling out interventions from one domain, there is a need for more evidence of packaged interventions because a combined set of interventions will likely result in the greatest improvement for adverse birth outcomes. Second, more research is needed to assess the longevity of interventions and its effectiveness across multiple life stages. For instance, only 17 out of 101 trials conducted follow-ups of women after birth delivery into the post-partum period. It is also important to note that the median follow-up of pregnant women beyond delivery was 8 weeks and only three trials ^23, 56, 57^ conducted follow-ups with women and their newborns up to 6 months of age.

Overall, we identified a number of interventions for pregnancy with clear and compelling supportive evidence for effectiveness for preventing adverse birth outcomes. In midst of the World Health Organization’s Global Nutrition Targets 2025 ^58^, which focuses on improving maternal, infant, and young children nutrition, national and local MNCH programs should consider adopting and adapting effective interventions identified in this review based on their local resource availability and program priorities. This may provide an opportunity to evaluate the benefits of these interventions in routine practice for pregnancy, and a step towards reaching the 2025 Global Nutrition Target of reducing the global prevalence of low birthweight by 30% ^59^.

## Data availability

### Underlying data

All data underlying the results are available as part of the article and no additional source data are required.

### Extended data

Open Science Framework: Interventions to improve birth outcomes of pregnant women living in low- and middle-income countries: a systematic review and network meta-analysis.

https://doi.org/10.17605/OSF.IO/JK3AQ ^19^.

This project contains the following extended data:

- Pregnancy NMA - Supplementary tables and figures - v2.0:
  - Appendix 1. Literature search strategy. (Contains Supplementary Tables 1–3.)
  - Appendix 2. Details of statistical analyses.
  - Appendix 3. List of included and excluded studies are full-text review. (Contains Supplementary Tables 4 and 5.)
  - Appendix 4. Details of the evidence base. (Contains Supplementary Tables 6 and 7.)
  - Appendix 5. Bias Assessment. (Contains Supplementary Table 8.)
  - Appendix 6. Intervention networks for birth outcomes (Supplementary Figures 1–6.)
  - Appendix 7. Primary analysis leverage and consistency plots. (Supplementary Figures 7–12.)
  - Appendix 8. Sensitivity analysis forest plots, non-cluster trials. (Supplementary Figures 13–15.)
  - Appendix 9. Sensitivity analysis leverage plots, non-cluster trials. (Supplementary Figures 16–18.)
- Pregnancy NMA - Supplementary crosstables - v1.0

### Reporting guidelines

Open Science Framework: PRISMA checklist for “Interventions to improve birth outcomes of pregnant women living in low- and middle-income countries: a systematic review and network meta-analysis.” https://doi.org/10.17605/OSF.IO/JK3AQ ^19^.

Data are available under the terms of the Creative Commons Attribution 4.0 International license (CC-BY 4.0).
