# Supplementary material for: Interventions to improve birth outcomes of pregnant women living in low- and middle-income countries: a systematic review and network meta-analysis
Source: Gates Open Res. 2020 Sep 24;3:1657. Originally published 2019 Nov 8. [Version 2] doi: 10.12688/gatesopenres.13081.2 (PMC7520556; doi:10.12688/gatesopenres.13081.2)
Supplement: Peer review history

# Peer review: Reviewer response for version 2

**DOI:** 10.21956/gatesopenres.14381.r29660

**Published:** 2020-09-25

**Authors:** R Rima Jolivet

**Affiliations:** 1 Women & Health Initiative, Harvard T.H. Chan School, Boston, MA, USA

I have no further comments and appreciate the authors' responses.

Are the rationale for, and objectives of, the Systematic Review clearly stated?

Partly

Is the statistical analysis and its interpretation appropriate?

I cannot comment. A qualified statistician is required.

Are sufficient details of the methods and analysis provided to allow replication by others?

Yes

Are the conclusions drawn adequately supported by the results presented in the review?

Partly

Reviewer Expertise:

Public health, maternal health, measurement, qualitative and quantitative research.

I confirm that I have read this submission and believe that I have an appropriate level of expertise to confirm that it is of an acceptable scientific standard.

# Peer review: Reviewer response for version 1

**DOI:** 10.21956/gatesopenres.14223.r29004

**Published:** 2020-07-16

**Authors:** Sonali Kochhar

**Affiliations:** 1 Department of Global Health, University of Washington, Seattle, WA, USA; 2 Global Healthcare Consulting, New Delhi, Delhi, India

- The comparative effectiveness of interventions across different domains to address adverse birth outcomes such as preterm birth, low birth weight, and birth weight for LMIC-based pregnant women is not known. This network meta-analysis study aims to compare interventions across domains like micronutrients, balanced energy protein supplements, maternal education, deworming, and WASH. Most clinical trials have compared within a domain, and summaries of the evidence for pregnancy interventions have used a pairwise meta-analysis to compare a single intervention with a comparator. Utilizing network meta-analysis, the study has prepared a network of interventions connected via the comparisons that have been made in head-to-head trials, with the interventions being compared indirectly via the common comparators.
- The study has been carefully designed and conducted and well reported. The results are not as significant as would have been expected. The author’s conclusions of the reasons for this are valid and include the limited existing evidence and low number of trials per comparison in the network.
- There are significant theoretical advantages of the network meta-analysis mentioned, but these are not clearly demonstrated in the study. The authors conclude that the findings of the study are generally similar to that of other existing reviews. The practical advantages of utilizing network meta-analysis in the study could be highlighted.
- It is not clear why some of the interventions were chosen as the common criteria are not immediately obvious. What was the maternal education about - nutrition or birth weight or?
- There was considerable variation in the enrollment of pregnant women in terms of the trimesters and gestational age. A number of studies involved women who were in their 2 ^nd^ and 3 ^rd^ trimesters. It is not expected that nutritional interventions in the later trimesters would have a significant impact on low birth weight or preterm birth. The authors acknowledge this. The authors could perhaps consider if there are there any other criteria that would be more beneficial to analyze.
- The authors mention that more research is needed to follow up women into the post-partum period. It would be useful if they substantiated this statement.
- This is a well-designed and conducted study. It would be useful if there was some thought was paid to the points mentioned above to increase the value of the study

Are the rationale for, and objectives of, the Systematic Review clearly stated?

Yes

Is the statistical analysis and its interpretation appropriate?

Yes

Are sufficient details of the methods and analysis provided to allow replication by others?

Yes

Are the conclusions drawn adequately supported by the results presented in the review?

Partly

Reviewer Expertise:

Maternal Immunization, Maternal and Child Research,  Adverse Maternal and neonatal outcomes

I confirm that I have read this submission and believe that I have an appropriate level of expertise to confirm that it is of an acceptable scientific standard, however I have significant reservations, as outlined above.

# Response

**Published:** 2020-08-20

**Authors:** Edward Mills

Dear Dr. Kochhar:

Thank you for your thorough review of our manuscript. Our responses to your recommendations and comments are marked with bullets below.  

Sincerely,

Edward J. Mills

The study has been carefully designed and conducted and well reported. The results are not as significant as would have been expected. The author’s conclusions of the reasons for this are valid and include the limited existing evidence and low number of trials per comparison in the network.

 

- *Thank you for the comment. We appreciate the time you took to review this article. *

 

There are significant theoretical advantages of the network meta-analysis mentioned, but these are not clearly demonstrated in the study. The authors conclude that the findings of the study are generally similar to that of other existing reviews. The practical advantages of utilizing network meta-analysis in the study could be highlighted.

 

- *Thank you for this comment. We have highlighted in the introduction and discussion sections that network meta-analysis allows the indirect comparison of multiple interventions connected via a common comparator when direct head-to-head evidence between interventions is not available. Since this direct evidence between interventions across multiple domains is very limited for pregnancy studies, we used network meta-analysis to compare the effectiveness of interventions across several domains. Figure 2 in the article demonstrates how the interventions are connected to each other via standard of care. Each line between the nodes represents a direct comparison between the interventions. Supplementary figures 1-6 demonstrate intervention networks for each outcome. Supplementary cross tables file provides effectiveness of different interventions domains relative to standard of care. Supplementary files can be found here (* *https://doi.org/10.17605/OSF.IO/JK3AQ* *). The forest plots provided in figures 3, 4, and 5 also demonstrate the comparative effectiveness of interventions relative to standard of care. *

 

It is not clear why some of the interventions were chosen as the common criteria are not immediately obvious. What was the maternal education about - nutrition or birth weight or?

 

- *The interventions were selected based on our PICOS criteria provided in Table 2 in page 5. The PICOS criteria was formulated based on existing reviews that have analyzed interventions for pregnant women. The list of existing reviews is provided in Table 1 in page 4.*
- *We have amended the results section to include the following: *

*“Maternal education was captured in only one trial where the education component was in the form of participatory learning action (PLA) with government-mandated women’s groups. PLA involved awareness of the problem of LBW and malnutrition, and strategies to overcome barriers to improved health and nutrition.” [Page 6, second column]*

There was considerable variation in the enrollment of pregnant women in terms of the trimesters and gestational age. A number of studies involved women who were in their 2nd and 3rd trimesters. It is not expected that nutritional interventions in the later trimesters would have a significant impact on low birth weight or preterm birth. The authors acknowledge this. The authors could perhaps consider if there are there any other criteria that would be more beneficial to analyze.

 

- *We have highlighted several areas of future research, such as looking at how combined set of interventions can impact adverse birth outcomes and assessing the longevity of interventions and its effectiveness across multiple life stages. *

 

The authors mention that more research is needed to follow up women into the post-partum period. It would be useful if they substantiated this statement.

 

- *We have indicated in the discussion section that only 17 out of 101 (16%) trials conducted follow-ups of women after delivery into the post-partum period. This is particularly important to assess the duration of effectiveness of interventions. This demonstrates the need to conduct more research in this area. *

 

This is a well-designed and conducted study. It would be useful if there was some thought was paid to the points mentioned above to increase the value of the study.

 

- *We are very thankful for your thorough review of our article and feedback. We hope that our responses sufficiently address the issues you have pointed out. *

# Peer review: Reviewer response for version 1

**DOI:** 10.21956/gatesopenres.14223.r29007

**Published:** 2020-07-14

**Authors:** Elizabeth M. McClure

**Affiliations:** 1 Social, Statistical and Environmental Health Sciences, RTI International, Durham, NC, USA

This is a novel approach to compare disparate interventions' impact on common adverse pregnancy outcomes. Most RCTs compare interventions within a domain in a common environment and most meta-analyses follow this approach. Attempting to compare the impact of interventions within different domains present methodologic challenges. The authors note an important limitation of the gestational age at which women were enrolled may have varied across these trials. Another potential factor that should be addressed is the potential for disparities in the populations as well as the environments. In particular, when comparing maternal nutritional interventions, understanding the effect of low BMI, maternal anemia and other relevant factors is important. Similarly, the environments, including access to high quality EMONC and other care can impact outcomes. It would be helpful if the authors could comment on these potential influences on their findings.

Are the rationale for, and objectives of, the Systematic Review clearly stated?

Yes

Is the statistical analysis and its interpretation appropriate?

Yes

Are sufficient details of the methods and analysis provided to allow replication by others?

Yes

Are the conclusions drawn adequately supported by the results presented in the review?

Yes

Reviewer Expertise:

Epidemiology, maternal and newborn health in LMICs

I confirm that I have read this submission and believe that I have an appropriate level of expertise to confirm that it is of an acceptable scientific standard, however I have significant reservations, as outlined above.

# Response

**Published:** 2020-08-20

**Authors:** Edward Mills

Dear Dr. McClure:

Thank you for your thorough review of our manuscript. Our responses to your recommendations and comments are marked with bullets below.  

Sincerely,

Edward J. Mills

This is a novel approach to compare disparate interventions' impact on common adverse pregnancy outcomes. Most RCTs compare interventions within a domain in a common environment and most meta-analyses follow this approach. Attempting to compare the impact of interventions within different domains present methodologic challenges. The authors note an important limitation of the gestational age at which women were enrolled may have varied across these trials. Another potential factor that should be addressed is the potential for disparities in the populations as well as the environments. In particular, when comparing maternal nutritional interventions, understanding the effect of low BMI, maternal anemia and other relevant factors is important. Similarly, the environments, including access to high quality EMONC and other care can impact outcomes. It would be helpful if the authors could comment on these potential influences on their findings.

 

- *Thank you for your comment. Network meta-analysis offers the advantage of making indirect comparison of multiple interventions connected via a common comparator. By employing network meta-analysis techniques, we were able to compare effectiveness of interventions across multiple domains.*
- *We acknowledge that there is substantial heterogeneity observed in the data. To address the heterogeneity in trials, we employed random-effects model for our network meta-analysis. It is also expected that nutritional interventions will positively impact maternal health parameters, such as BMI and anemia which in turn will reduce the incidence of adverse birth outcomes. External factors, such as access to quality EMONC and other care settings are outside the scope of our research article. *

# Peer review: Reviewer response for version 1

**DOI:** 10.21956/gatesopenres.14223.r28944

**Published:** 2020-07-10

**Authors:** R Rima Jolivet

**Affiliations:** 1 Women & Health Initiative, Harvard T.H. Chan School, Boston, MA, USA

- This is a carefully conducted study with great attention to study quality, using and diligently reporting best available criteria for quality in meta analytic studies. The results are somewhat less significant than could have been hoped for given the breadth of the search and network analytic design. I agree largely with the authors’ assessment of the study limitations, which mostly have to do with limitations in the existing evidence base.
- While I have not had enough volume or very recent experience conducting meta analyses to feel overly confident critiquing the statistical or interpretational decisions made, I do have some relatively minor questions about the design and the conclusions.
- I am not familiar with the network meta analytic methodology; however, it is hard to see the added value afforded by the network approach, since there is no discussion of intersectionality, synergies, or correlations between domains of intervention. Thus, the additional value afforded by the network meta analysis is never clearly described. This may be because the underlying studies did not allow for such comparison, but the description of the methodology suggests that it would allow the researchers to take single intervention RCTs and combine their results to evaluate a web of effects. That does not come through in the paper. Despite this, the authors list the methodology as the paper’s greatest strength.
- The authors state, “The purpose of this study was to assess the comparative effectiveness across intervention domains in micronutrient supplements, balanced energy protein supplements, deworming, maternal education, and WASH interventions…”  In addition to the previous comment, I wondered on what basis the interventions were selected, as a unifying hypothesis was not given. The studies listed include several whose interventions have nothing to do with nutrition (financial incentives; flu immunization), and it was not clear why they were included. In addition, the authors do not specify whether maternal education, one of the intervention domains, was education specific to nutrition, or to preterm birth and birthweight? If not, it is unclear how the educational intervention serves as a comparator or how it relates to the nutritional interventions listed in the study aims.
- The authors state that the majority of trials recruited women who were in the later trimesters (n=69). This is potentially significant, given that nutritional determinants of preterm birth and low birth weight are likely to have their origin well before the time of study enrollment, and it is not surprising that the effects of nutritional interventions may not be observed in such a short period of time. This limitation is acknowledged by the authors. However, it raises the question of whether additional outcomes more likely to show a difference, such as mean difference in Hgb and Hct, might have been good to explore.
- While I agree largely with the authors’ assessment of the study limitations and commend them for their attention to quality, and for adjusting for the clustering effects and choosing a conservative ICC, I differ with some points made in the Discussion section. The authors state that, “The main strength of this study was the use of network meta analysis to assess the effectiveness of different interventions from a large network of evidence compared to standard-of-care.” However, the value of this approach and what it showed compared to a simple meta analysis was not adequately demonstrated and could perhaps be further emphasized.
- The authors state that “there is a need for more evidence of packaged interventions because a combined set of interventions will likely result in the greatest improvement for adverse birth outcomes” but they do not present any evidence for this hypothesis in the current study, as would be expected based on their assertions about network meta analysis. There does not appear to be any statistical analysis that shows that combined interventions resulted in a greater effect size or better outcomes. 
- Finally, the discussion of the lack of postpartum follow-up with women (while valuable in its own right) is also hard to understand in the context of this study whose outcomes focused on the effects of maternal nutrition in pregnancy on gestational age and birthweight, since there were no outcomes related to the postnatal period for mothers or newborns.
- Overall, this is a well conducted study, that reflects possibly some areas for improvement in the description of the study aims, justification of the choice of methodology, and interpretation of the results (factors within the author’s control), and well as limitations in the underlying evidence base (factors outside the authors’ control).

 

Other minor comments:

- Exclusion of non-English pubs could be significant given the target of LMIC populations.
- The authors state, “All reviews were systematic literature reviews with pairwise meta-analysis, except for Glassman 2013 and Yang 2011”, but do not clarify what kind of studies these two were.
- I was unable to find the network diagrams and thus could not evaluate this aspect of the study.  The narrative in the paper does not seem to adequately address the elements of synergy/relationship between the intervention domains, or the lack thereof found through the analysis.
- The reported relative risk for preterm birth for iron supplementation contains 1: (iron: RR=0.70, 95% credible interval [Crl] 0.47, 1.01).
- I appreciate that the authors performed adjustments for the clustering effect of cluster randomization as well as sensitivity analyses to remove those trials, since other independent variables come into play in results observed with cluster randomization vs individual allocation. This study demonstrates the reason why individual allocation is valuable for exploring effectiveness of interventions whose mechanism of action is at the person level.
- The fact that the authors’ search uncovered no individually randomized trials that evaluated the effectiveness of high caloric (1500 kcal per day) local food for pregnant women on birth outcomes demonstrates the problem that funder-driven trends in research design pose. The lack of nuance in the preference for implementation research aimed at scaling up interventions contributes to an inability to evaluate the effectiveness of potentially impactful interventions on one hand, and to the spread of interventions unproven at scale.  This is not the within the authors’ scope of responsibility but it is an important finding.
- The interventions described in the opening to the Discussion should match those described in the research question, but do not (the Discussion describes “several domains ranging from nutrition, infection control, and education”). This raises the question again of the underlying framework or hypothesis guiding the selection of intervention domains (is it nutrition or broader?), and if broader what kinds of correlation or interaction a network analysis was expected to show.
- I wonder why the effect of the 1500 kcal local food supplement is de-emphasized, since a statistically significant RR of 0.17 for preterm birth seems like it would be worth highlighting more than the RR = 0.70 for iron that contains 1.

Are the rationale for, and objectives of, the Systematic Review clearly stated?

Partly

Is the statistical analysis and its interpretation appropriate?

I cannot comment. A qualified statistician is required.

Are sufficient details of the methods and analysis provided to allow replication by others?

Yes

Are the conclusions drawn adequately supported by the results presented in the review?

Partly

Reviewer Expertise:

Public health, maternal health, measurement, qualitative and quantitative research.

I confirm that I have read this submission and believe that I have an appropriate level of expertise to confirm that it is of an acceptable scientific standard, however I have significant reservations, as outlined above.

# Response

**Published:** 2020-08-20

**Authors:** Edward Mills

Dear Dr. Jolivet:

Thank you for these thorough reviews of our manuscript. Our responses to your recommendations and comments are marked with bullets below.  

Sincerely,

Edward J. Mills

This is a carefully conducted study with great attention to study quality, using and diligently reporting best available criteria for quality in meta analytic studies. The results are somewhat less significant than could have been hoped for given the breadth of the search and network analytic design. I agree largely with the authors’ assessment of the study limitations, which mostly have to do with limitations in the existing evidence base. While I have not had enough volume or very recent experience conducting meta analyses to feel overly confident critiquing the statistical or interpretational decisions made, I do have some relatively minor questions about the design and the conclusions.

I am not familiar with the network meta analytic methodology; however, it is hard to see the added value afforded by the network approach, since there is no discussion of intersectionality, synergies, or correlations between domains of intervention. Thus, the additional value afforded by the network meta analysis is never clearly described. This may be because the underlying studies did not allow for such comparison, but the description of the methodology suggests that it would allow the researchers to take single intervention RCTs and combine their results to evaluate a web of effects. That does not come through in the paper. Despite this, the authors list the methodology as the paper’s greatest strength.

 

- *Thank you for this comment. Network meta-analysis allows the indirect comparison of multiple interventions connected via a common comparator. Given that there is a scarcity of direct head-to-head randomized clinical trials (RCTs) between existing interventions in pregnancy, we felt this analytical approach was the most optimal way to summarize the current evidence base for this important life-stage. Figure 2 in the article demonstrates how the interventions are connected to each other via standard of care. Each line between the nodes represents a direct comparison between the interventions. Supplementary figures 1-6 demonstrate intervention networks for each outcome. Moreover, under the results section from pages 7-9, we discussed how some of the interventions impact the outcomes of interest. Forrest plots have also been provided to show the effects of interventions on our outcomes of interest. *

 

The authors state, “The purpose of this study was to assess the comparative effectiveness across intervention domains in micronutrient supplements, balanced energy protein supplements, deworming, maternal education, and WASH interventions…”  In addition to the previous comment, I wondered on what basis the interventions were selected, as a unifying hypothesis was not given. The studies listed include several whose interventions have nothing to do with nutrition (financial incentives; flu immunization), and it was not clear why they were included.

 

- *We apologize for the confusion. We have removed mention of the studies related to financial incentives and flu immunization in Table 1. [New version of the manuscript has been submitted to Gates Open Research]*
- *We selected the scope of the interventions based on existing reviews that have analyzed interventions for pregnant women based on the hallmark paper by Bhutta 2013 (Bhutta ZA, Das JK, Rizvi A, Gaffey MF, Walker N, Horton S, Webb P, Lartey A, Black RE, Group TL, Maternal and Child Nutrition Study Group. Evidence-based interventions for improvement of maternal and child nutrition: what can be done and at what cost? The Lancet. 2013 Aug 3;382(9890):452-77.). We have updated the manuscript to provide justifications of our study scope. *

 " ***The scope of our research study and the corresponding search strategy*** *was developed after first reviewing the papers published in the Lancet’s 2013 Maternal and Child Nutrition series, including the umbrella review on evidence-* *based interventions by Bhutta and colleagues, for an overview of the literature*."

In addition, the authors do not specify whether maternal education, one of the intervention domains, was education specific to nutrition, or to preterm birth and birthweight? If not, it is unclear how the educational intervention serves as a comparator or how it relates to the nutritional interventions listed in the study aims.

 

- *There was only one study pertaining to maternal education (https://pubmed.ncbi.nlm.nih.gov/29742136/) which also included a combination of financial incentive program to encourage pregnant mothers to buy food. Description of intervention domains for each included study has been provided in Supplementary table 7. We have not included any study that involves flu immunization. *
- *We have amended the results section to include the following: “Maternal education was captured in only one trial where the education component was in the form of participatory learning action (PLA) with government-mandated women’s groups. PLA involved awareness of the problem of LBW and malnutrition, and strategies to overcome barriers to improved health and nutrition.” [Page 6, second column]*

 

The authors state that the majority of trials recruited women who were in the later trimesters (n=69). This is potentially significant, given that nutritional determinants of preterm birth and low birth weight are likely to have their origin well before the time of study enrollment, and it is not surprising that the effects of nutritional interventions may not be observed in such a short period of time. This limitation is acknowledged by the authors. However, it raises the question of whether additional outcomes more likely to show a difference, such as mean difference in Hgb and Hct, might have been good to explore.

 

- *Thank you for this comment. The decision to limit the scope of our study to preterm birth, low birthweight and mean birthweight outcomes was done based on our initial review of Bhutta et al 2013 and other important reviews during the project planning stage. While other outcomes such as Hgb and Hct are undoubtedly important, this is beyond the scope of our study.*

 

While I agree largely with the authors’ assessment of the study limitations and commend them for their attention to quality, and for adjusting for the clustering effects and choosing a conservative ICC, I differ with some points made in the Discussion section. The authors state that, “The main strength of this study was the use of network meta analysis to assess the effectiveness of different interventions from a large network of evidence compared to standard-of-care.” However, the value of this approach and what it showed compared to a simple meta analysis was not adequately demonstrated and could perhaps be further emphasized.

- *We have pointed out in the methods section that traditional pairwise meta-analysis only offers quantitative assessment of a single intervention versus a comparator. In network meta-analysis, we were able to strengthen the evidence base by analyzing both direct and indirect evidence collectively. Other existing reviews that have conducted a pairwise meta-analysis have pooled cluster and individually randomized clinical trials without adjusting for the intracluster correlation, so their effect estimates were heavily influenced by the cluster randomized clinical trials that were generally larger in sample size. While the most of cluster randomized clinical trials did not report their ICC value, this approach of pooling these two types of clinical trials is clearly inappropriate. We were forced to make an assumption of 0.05 ICC. We have acknowledged that this is a limitation while being more appropriate than previous analytical approaches that have been used for existing reviews.*

 

The authors state that “there is a need for more evidence of packaged interventions because a combined set of interventions will likely result in the greatest improvement for adverse birth outcomes” but they do not present any evidence for this hypothesis in the current study, as would be expected based on their assertions about network meta analysis. There does not appear to be any statistical analysis that shows that combined interventions resulted in a greater effect size or better outcomes.

 

- *Thank you for your comment. We have demonstrated through random effects network meta-analysis models that several micronutrient supplements decreased risks of pre-term birth and improved mean birthweight, compared to standard of care. Estimates from these analyses are provided in the forest plots as well as the cross-tables in the supplementary file. We have also pointed out in the discussion section that while network meta-analysis allows us to compare interventions indirectly across multiple domains, due to limited evidence base, our analyses was limited as well. The evidence base consisting of trials that report only on a single domain of intervention is a big limiting factor. *

 

Finally, the discussion of the lack of postpartum follow-up with women (while valuable in its own right) is also hard to understand in the context of this study whose outcomes focused on the effects of maternal nutrition in pregnancy on gestational age and birthweight, since there were no outcomes related to the postnatal period for mothers or newborns.

 

- *Thank you for this comment. We highlighted the need for follow-ups of women after birth delivery into the post-partum period in order to provide future direction of research to assess the longevity of interventions and its effectiveness across multiple life stages. *

 

Overall, this is a well conducted study, that reflects possibly some areas for improvement in the description of the study aims, justification of the choice of methodology, and interpretation of the results (factors within the author’s control), and well as limitations in the underlying evidence base (factors outside the authors’ control).

 

- *Thank you for this compliment. *

 

Exclusion of non-English pubs could be significant given the target of LMIC populations.

 

- *While we acknowledge that inclusion of non-English studies will reduce bias in reviews, including non-English studies will require services of professional translators, and the research process will become costly and time-consuming as a result. *

 

The authors state, “All reviews were systematic literature reviews with pairwise meta-analysis, except for Glassman 2013 and Yang 2011”, but do not clarify what kind of studies these two were.

 

- *Table 1 provides details, such as title, interventions used, types of studies included, of existing reviews on pregnancy interventions. We have removed Glassman 2013 from the table. Description of Yang 2011 is provided in Table 1.*

 

I was unable to find the network diagrams and thus could not evaluate this aspect of the study.  The narrative in the paper does not seem to adequately address the elements of synergy/relationship between the intervention domains, or the lack thereof found through the analysis.

 

- *The Supplementary tables and figures file can be found here:* *https://doi.org/10.17605/OSF.IO/JK3AQ* *(Click on Files section)* *. Networks diagrams for birth outcomes are provided in supplementary figures 1-6. Supplementary cross tables file provides effectiveness of different interventions domains relative to standard of care. Figure 2 in the article demonstrates how the interventions are related to one another through standard of care. *

 

 The reported relative risk for preterm birth for iron supplementation contains 1: (iron: RR=0.70, 95% credible interval [Crl] 0.47, 1.01).

- *This is correct which is why we interpreted this as a trend towards lower preterm birth risks compared to standard of care but the Crl overlapped the null effect of 1. *

 I appreciate that the authors performed adjustments for the clustering effect of cluster randomization as well as sensitivity analyses to remove those trials, since other independent variables come into play in results observed with cluster randomization vs individual allocation. This study demonstrates the reason why individual allocation is valuable for exploring effectiveness of interventions whose mechanism of action is at the person level.

- *We thank you for this feedback.*

 

The fact that the authors’ search uncovered no individually randomized trials that evaluated the effectiveness of high caloric (1500 kcal per day) local food for pregnant women on birth outcomes demonstrates the problem that funder-driven trends in research design pose. The lack of nuance in the preference for implementation research aimed at scaling up interventions contributes to an inability to evaluate the effectiveness of potentially impactful interventions on one hand, and to the spread of interventions unproven at scale.  This is not the within the authors’ scope of responsibility but it is an important finding.

- *We agree with you. While beyond the scope of this study, you are absolutely correct in your assertion here.*

The interventions described in the opening to the Discussion should match those described in the research question, but do not (the Discussion describes “several domains ranging from nutrition, infection control, and education”). This raises the question again of the underlying framework or hypothesis guiding the selection of intervention domains (is it nutrition or broader?), and if broader what kinds of correlation or interaction a network analysis was expected to show.

 

- *Thank you for this comment. We have amended the discussion section to include the following:*

" *We used network meta-analysis to compare the effectiveness of interventions across several domains **consisting of micronutrient supplements, balanced energy protein supplements, deworming, maternal education, and WASH interventions** that can be provided to pregnant women living in LMICs”. [Page 10, 2 ^nd^ column]*

 

- *Our objective was to compare the effectiveness of interventions not only involving nutrition but also deworming and WASH in impacting birth outcomes in LMICs. Due to limited evidence base, we didn’t find any concrete findings in regards to how maternal education and deworming affected birth outcomes. And there were no WASH trials that reported on our outcomes of interest. *

 

I wonder why the effect of the 1500 kcal local food supplement is de-emphasized, since a statistically significant RR of 0.17 for preterm birth seems like it would be worth highlighting more than the RR = 0.70 for iron that contains 1.

 

- *We have mentioned both under results and discussion sections that consumption of 1500 kcal of local food supplement lowered the risks of preterm birth and low birthweight. For iron (RR: 0.70, 95% CrI: 0.47, 1.01), we have indicated that it showed a trend towards lower preterm birth risks compared to standard-of-care, but the Crl overlapped the null effect of 1.00.*
